# Amorphous Vanadium Oxide Thin Films as Stable Performing Cathodes of Lithium and Sodium-Ion Batteries

**DOI:** 10.1186/s11671-018-2766-0

**Published:** 2018-11-14

**Authors:** Shaikshavali Petnikota, Rodney Chua, Yang Zhou, Eldho Edison, Madhavi Srinivasan

**Affiliations:** School of Materials Science and Engineering, Nanyang Technological University, Singapore, 639798 Singapore

**Keywords:** Amorphous vanadium oxide, PLD, Thin films, Cathode, Li- and Na-ion batteries

## Abstract

Herein, we report additive- and binder-free pristine amorphous vanadium oxide (a-VOx) for Li- and Na-ion battery application. Thin films of a-VOx with a thickness of about 650 nm are grown onto stainless steel substrate from crystalline V_2_O_5_ target using pulsed laser deposition (PLD) technique. Under varying oxygen partial pressure (pO_2_) environment of 0, 6, 13 and 30 Pa, films bear O/V atomic ratios 0.76, 2.13, 2.25 and 2.0, respectively. The films deposited at 6‑30 Pa have a more atomic percentage of V^5+^ than that of V^4+^ with a tendency of later state increased as pO_2_ rises. Amorphous VOx films obtained at moderate pO_2_ levels are found superior to other counterparts for cathode application in Li- and Na-ion batteries with reversible capacities as high as 300 and 164 mAh g^−1^ at 0.1 C current rate, respectively. At the end of the 100th cycle, 90% capacity retention is noticed in both cases. The observed cycling trend suggests that more is the (V^5+^) stoichiometric nature of a-VOx better is the electrochemistry.

**Electronic supplementary material:**

The online version of this article (10.1186/s11671-018-2766-0) contains supplementary material, which is available to authorized users.

## Introduction

Amorphous vanadium oxides (a-VOx) are becoming increasingly popular for the application of electronic devices [1, 2], secondary lithium and post-lithium-ion batteries [3–13]. Compared to crystalline counterparts, a-VOx could offer shorter diffusion paths and, in turn, easy lithiation kinetics that facilitates achieving theoretical capacities in practical conditions. Crystalline oxides such as V_2_O_5_ possess the theoretical capacity of lithium intake up to 441 mAh g^−1^ (1 C). In practice, this theoretical capacity accounts for 3 mol of Li that needs to be discharged to 1.5 V. But the formation of irreversible ω-Li_x_V_2_O_5_ phase when discharged below 1.9 V limits the reversible capacity to 2 mol of Li only [8]. For this reason, crystalline V_2_O_5_ must be cycled within a voltage window of 4.0–2.0 V that corresponds to theoretical capacity 292 mAh g^−1^. Hence, despite the huge theoretical capacity, crystalline vanadium oxides exhibit lower experimental capacities. In this context, a-VOx materials emerged as high capacity cathodes that are free from irreversible phase formations and thus overcoming the operating voltage constraint [5, 8]. For example, sol-gel synthesised amorphous V_2_O_5_·*x*H_2_O (*x* = 2–2.3) exhibited a high reversible capacity of 410 mAh g^−1^ at a C/8 current rate [14]. In a recent report, electrodeposited a-VOx film as a cathode of Na-ion battery (SIB) performed better than their crystalline counterpart with double the capacity (241 mAh g^−1^) [10]. However, in these two cases, cycling stability is not good. In the late 1990s, Zhang et al. and McGraw et al. reported a-VOx films prepared by pulsed laser deposition (PLD) with specific capacity of 346 mAh g^−1^ at 100th cycle in the voltage range of 1.5–4.1 V and at a current rate of 0.1 mA cm^−2^ [3, 4]. However, in these cases, the collective contribution of the conductive SnO_2_ substrate that is electrochemically active to the high capacity remains unknown. The film of a-VOx about 450 nm thick prepared by atomic layer chemical vapour deposition (ALCVD) touted good cycling stability at 0.1 C rate with reversible capacity of 275 mAh g^−1^ at the end of the 100th cycle [6]. A 30-nm-thick a-VOx film grown by ALD exhibited superior cycling performance than afore-discussed amorphous materials with a 100th cycle capacity of 330 mAh g^−1^ at 1 C rate in-between 1.5 and 4.0 V [7]. Similar cycling behaviour was noticed for quasi-amorphous VOx films deposited by ALD with a capacity around 60 mAh g^−1^ at 1 C rate in the voltage range of 2.75–3.8 V [14]. In a recent study, a-VOx electrochemically deposited onto graphite paper as a cathode for sodium-ion battery showed superior cyclability than the crystalline counterpart at lower specific currents in the range of 80–320 mA g^−1^ [11]. Therefore, amorphous vanadium oxides are superior to the crystalline counterpart for the electrochemical energy storage applications. However, to the best of our knowledge, there is no comprehensive study regarding the cathodic functioning of pristine a-VOx that extensively correlates the performance with key fundamental properties such as the extent of amorphization, stoichiometry and oxygen to vanadium coordination. Such a study was initiated by Julien et al. in 1999 to get amorphous V_2_O_5_ thin films by PLD at pO_2_ ~ 13.33 and 19.995 Pa and at a substrate temperature of 300 °C that is too high to get the amorphous phase [15]. At this temperature, amorphous V_2_O_5_ phase formation is not likely possible as it is well above 200 °C which is widely agreed minimum temperature limit to get the crystalline phase [16–21]. The detailed elemental and chemical (XPS) composition analysis of the so claimed amorphous V_2_O_5_ phase needs to be studied. The presented lithiation electrochemistry of V_2_O_5_ deposited at 300 °C on glass or silicon substrates confirms that films were indeed crystalline by visualizing clear long-lasting (~ 35 h) Li-intercalation plateaus around 2.6 V. In addition, presented electrochemistry measured over perfect insulating natured Si and glass (including V_2_O_5_ film) substrates without proper current collector pose ambiguity in considering it to compare with other similar works. However, in comparison to the present PLD a-VOx study, there was no lithiation or sodiation electrochemistry of the amorphous phase was discussed. Hence, we opted to study pulsed laser-deposited a-VOx films grown at different oxygen partial pressures (pO_2_) for lithium-ion and sodium-ion battery applications.

Physical and chemical nature of the PLD films can be easily tailored by controlling the reactive oxygen gas. A systematic study of such controlling parameters and the consequences on the final property are in due to report. Such a study is very important for exploring the fundamental aspects as a function of varying oxygen stoichiometry. Pulsed laser deposition is one of the best ways to study the fundamental electrochemical properties of a-VOx in their pristine state. Additionally, there is no need for additives such as carbon and binder. Furthermore, chemical impurities like water molecules occupied lattice sites or surface hydroxyls that emerge during wet chemical synthesis process could be avoided through PLD. Moreover, the chemical impurities could cause considerable capacity loss or pose ambiguity in exact electrochemical characteristics. For example, electrodeposited a-VOx·*n*H_2_O [10], a-V_2_O_5_ [11] and melt-quenched V_2_O_5_·*n*H_2_O xero-gel [22] compounds as cathodes of SIBs exhibited totally different and asymmetric sodium insertion and desertion features than in the present study. Moreover, a-VOx·*n*H_2_O [10] and V_2_O_5_·*n*H_2_O [22] compounds delivered high initial capacities that rapidly decayed to the stable capacities obtained throughout the cycling in the present study. Such a rapid decay in capacity might plausibly arise from the interaction of hydroxyl species with Na-ions and/or with electrolyte molecules to form irreversible covalent compounds. Therefore, in this present study, a-VOx films prepared by PLD are investigated for their physical and chemical properties in correlation with lithiation and sodiation electrochemistry. The obtained results are compared with available literature as well as with commercial V_2_O_5_ bulk powder whose electrodes were made by adding carbon and binder.

## Methods

### Thin Film Deposition

Vanadium oxide films were deposited using crystalline V_2_O_5_ as a target by PLD. High-energy KrF excimer laser (*λ* = 248 nm) was focused on the surface of V_2_O_5_ target, with a power of ~ 200 mJ and repetition of 5 Hz. Amorphous VOx films were grown on 304-grade stainless steel (SS) substrate preheated to 100 °C and under different oxygen partial pressures (pO_2_). The substrate-target distance was kept at ~ 5 cm. The deposition was carried out under a high vacuum of 0.6 × 10^−5^ mbar at first, and then oxygen gas was introduced equivalent to pO_2_ ~ 6, 13 and 30 Pa. Each film was deposited for 44 min that resulted in approximately 650 nm thickness. For forthcoming discussion, these four types of films are abbreviated as a-VOx-0 Pa, a-VOx-6 Pa, a-VOx-13 Pa and a-VOx-30 Pa.

### Characterization

Electron micrographs and elemental composition (EDAX) of the films were obtained with JEOL 7600F field emission scanning electron microscope (FESEM) that operated at 5 kV acceleration voltage. Structural and phase characterization was carried out with Bruker D8 Advance XRD with Cu-Ka radiation (*λ* = 1.54 Å) that operated at 40 kV and 40 mA. The atomic force microscope (AFM) images were obtained from a commercial AFM (Asylum Research MFP3D), by using Asylum Research AC240TM tips (Pt/Ti-coated, 70 kHz and 2 N/m). X-ray photoelectron spectroscopy (XPS) was performed with a monochromatic Mg X-ray radiation source in a Phoibos spectrometer (SPECS, Germany). High-resolution survey spectra were analysed with Casa XPS software package.

### Electrochemical Characterization

As-deposited films were used as cathodes to fabricate coin cells CR2016 with Li and Na metal as the counter electrodes. Whatman glass microfiber filter paper was used as the separator. One molar LiPF_6_ in ethylene carbonate (EC) and diethyl carbonate (DEC) (1:1 by volume) was used as the electrolyte for Li-ion batteries (LIBs). One molar NaClO_4_ in propylene carbonate (PC) with 5% fluoroethylene carbonate (FEC) was used as electrolyte to fabricate SIBs. Coin cell fabrication was carried out inside a glove box filled with Ar gas where moisture and oxygen levels strictly limited to less than 0.1 ppm.

Battery testing was carried out at room temperature after 8 h of relaxation. The cyclic voltammetry (CV) measurements for both LIBs and SIBs were carried out in the voltage range of 1.5–4.0 V at a scan rate of 0.1 mV s^−1^ using SOLARTRON 1470E equipment. The galvanostatic charge-discharge (GC) tests were carried out in the same voltage range as in the case of CV measurements and at a current rate of 0.1 to 10.0 C using NEWARE battery test system. Here, 1 C = 294 and 441 mA g^−1^ for voltage windows 2.0–4.0 and 1.5–4.0 V, respectively, for LIBs. In case of SIBs, 1 C = 236 mA g^−1^ for a voltage range of 1.5–4.0 V. Electrochemical impedance spectroscopy (EIS) measurements were carried out in the frequency range of 100,000–0.01 Hz and with a voltage amplitude of 10 mV using Frequency Response Analyser, Solartron Analytical 1400 CellTest System.

## Results and Discussion

### Physical and Chemical Characterization

**Fig. 1:**
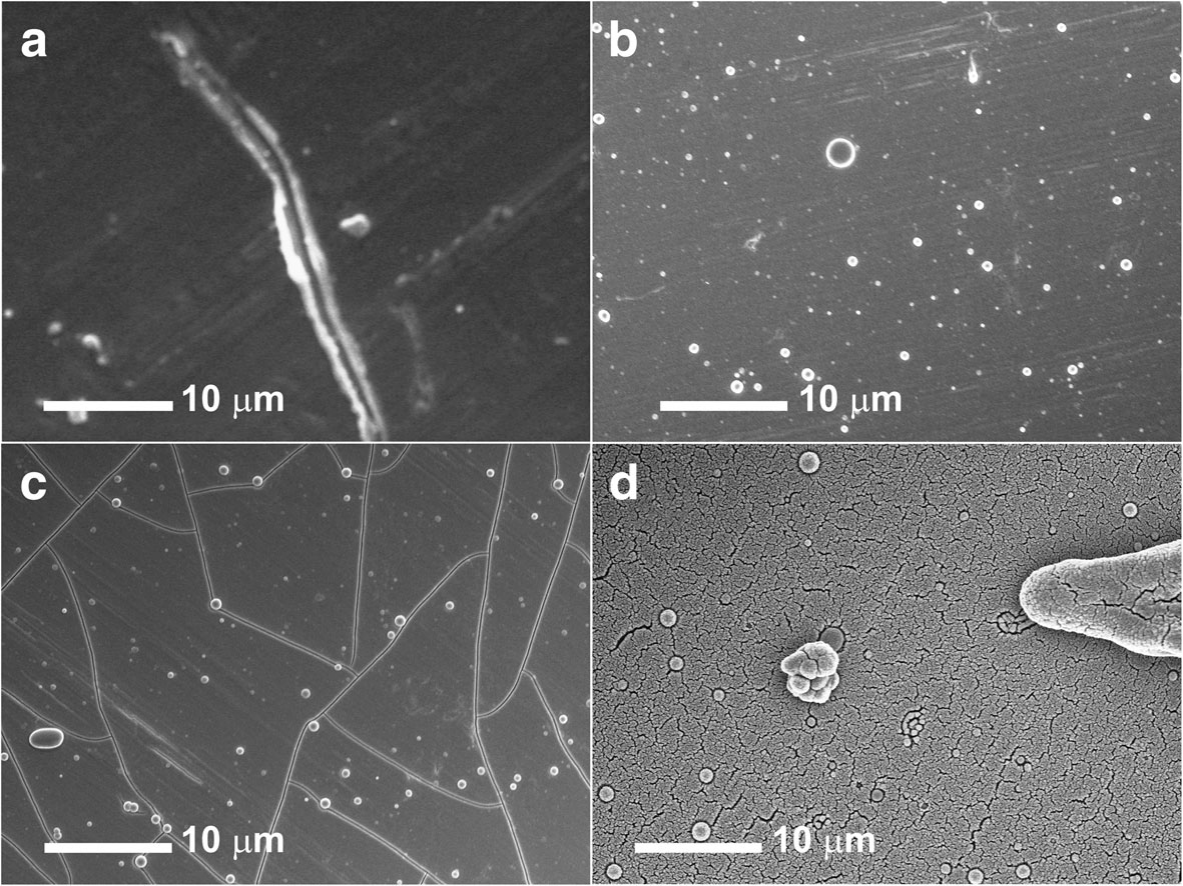
Field emission scanning electron micrographs of a-VOx films deposited at pO_2_ (**a**) 0 Pa, (**b**) 6 Pa, (**c**) 13 Pa and (**d**) 30 Pa

Physical morphology of the as-deposited films observed under FESEM is as shown in Fig. 1. Film a-VOx-0 Pa (Fig. 1a) is smooth and continuous but resembled bare stainless steel substrate (Additional file 1: Figure S11) possessing trenches on the surface. In Fig. 1b, a-VOx-6 Pa film is also found to be continuous but with lots of accumulated spherical particulates. Similar features are observed in the case of a-VOx-13 Pa film (Fig. 1c) but is totally different from the other two films by having large grain boundaries several tens of microns in size. In common, these three films are highly transparent to electron beam as scratch marks on bare stainless steel are clearly discernable. In contrast to these three films, a-VOx-30 Pa is opaque and non-continuous (Fig. 1d). It possesses very small submicron-sized grain boundaries and very large randomly shaped particulates at the order of 10 μm in length. In typical PLD system, high-energy laser strikes on the surface of the target and causes the evaporation of the target materials, which forms the active plume (i.e. the mixture of energetic species such as atoms, molecules, electrons and ions) and then the growth of thin films on the substrates. During deposition, pO_2_ highly affects the collision and reaction between the plume and the oxygen atoms, and thus the shape and size of the plume. Therefore, the growth mode, deposition rate and the homogeneity of the thin film depend on pO_2_ [23, 24]. In general, higher pO_2_ leads to attenuation and slowing of the plume, resulting in larger grain size and rougher film [25]. In our case, when pO_2_ is fixed at 30 Pa, the roughness of the VOx film is too large (non-reflective) and some of the grains are isolated, and hence, the film featured opaque and non-continuous topography. The elemental compositions of a-VOx films obtained by EDAX technique are enumerated in Table 1. Film deposition under vacuum resulted in very poor O/V atomic ratio, and for this reason, a-VOx-0 Pa film was omitted from further analysis. Next, the O/V ratio is increased to 2.135 with the introduction of oxygen into the deposition chamber that is equivalent to 6 Pa. As shown in Table 1, there is no significant increase in the O/V ratio even after doubling oxygen to 13 Pa. Further increase in oxygen to a very high level of 30 Pa lowered the O/V ratio to 2.003. The unchanged or decreased O/V ratio might have plausibly taken place from a change in the transport property such as reduction in mean free path (*λ*) of the oxygen species with increasing pO_2_ (*λ α* 1/pO_2_) [26].

**Table 1:** Elemental composition of a-VOx films

Film	O (At%)	V (At%)	O/V ratio
a-VOx-0 Pa	43.1	56.9	0.757
a-VOx-6 Pa	68.1	31.9	2.135
a-VOx-13 Pa	69.2	30.8	2.247
a-VOx-30 Pa	66.7	33.3	2.003

**Fig. 2:**
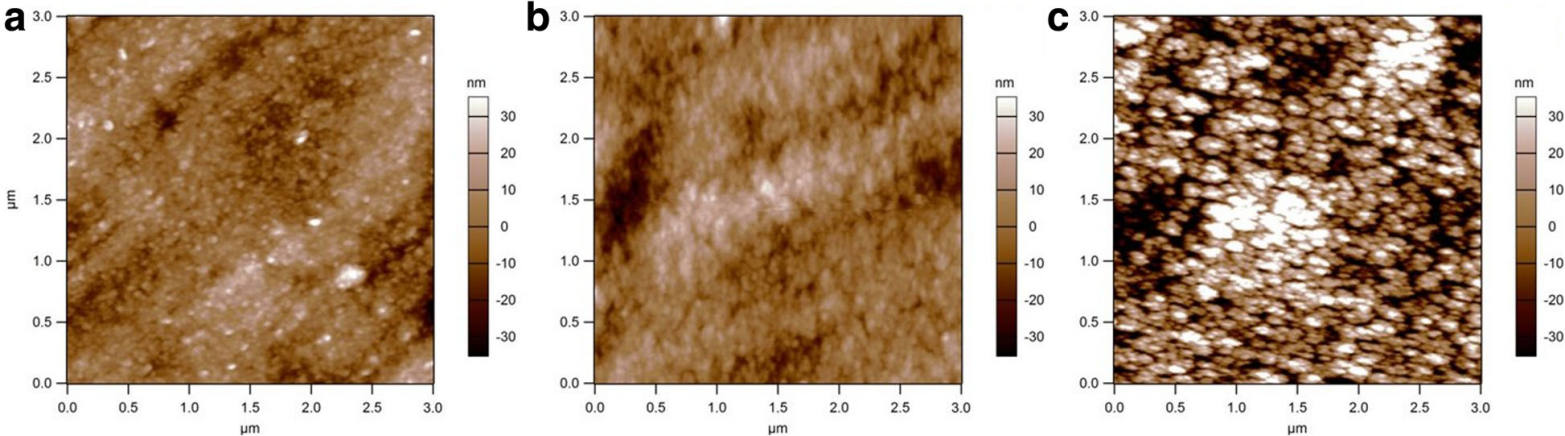
AFM surface topography of a-VOx-6 Pa (**a**), a-VOx-13 Pa (**b**) and a-VOx-30 Pa (**c**)

XRD patterns of all the films are similar and contained peaks corresponding to stainless steel only as shown in Additional file 1: Figure S12. This clearly confirms that all the films are solely amorphous. AFM surface topography of a-VOx films as shown in Fig. 2 is well complying with the physical morphology observed in electron micrographs (Fig. 1). Films a-VOx-6 Pa (Fig. 2a) and a-VOx-13 Pa (Fig. 2b) bear similar surface features and smooth in accordance with their respective electron micrographs. On the other hand, the surface of a-VOx-30 Pa (Fig. 2c) film is very rough and uneven in contrast to the other two films. Measured average roughness over a 3 × 3 μm scanning is found to be ~ 8.6, ~ 9.2 and ~ 24 nm for a-VOx-6 Pa, a-VOx-13 Pa and a-VOx-30 Pa films, respectively. Therefore, an increase in oxygen partial pressure not only increases the surface roughness but also induces a change in morphology as observed in the FESEM micrographs.

**Fig. 3:**
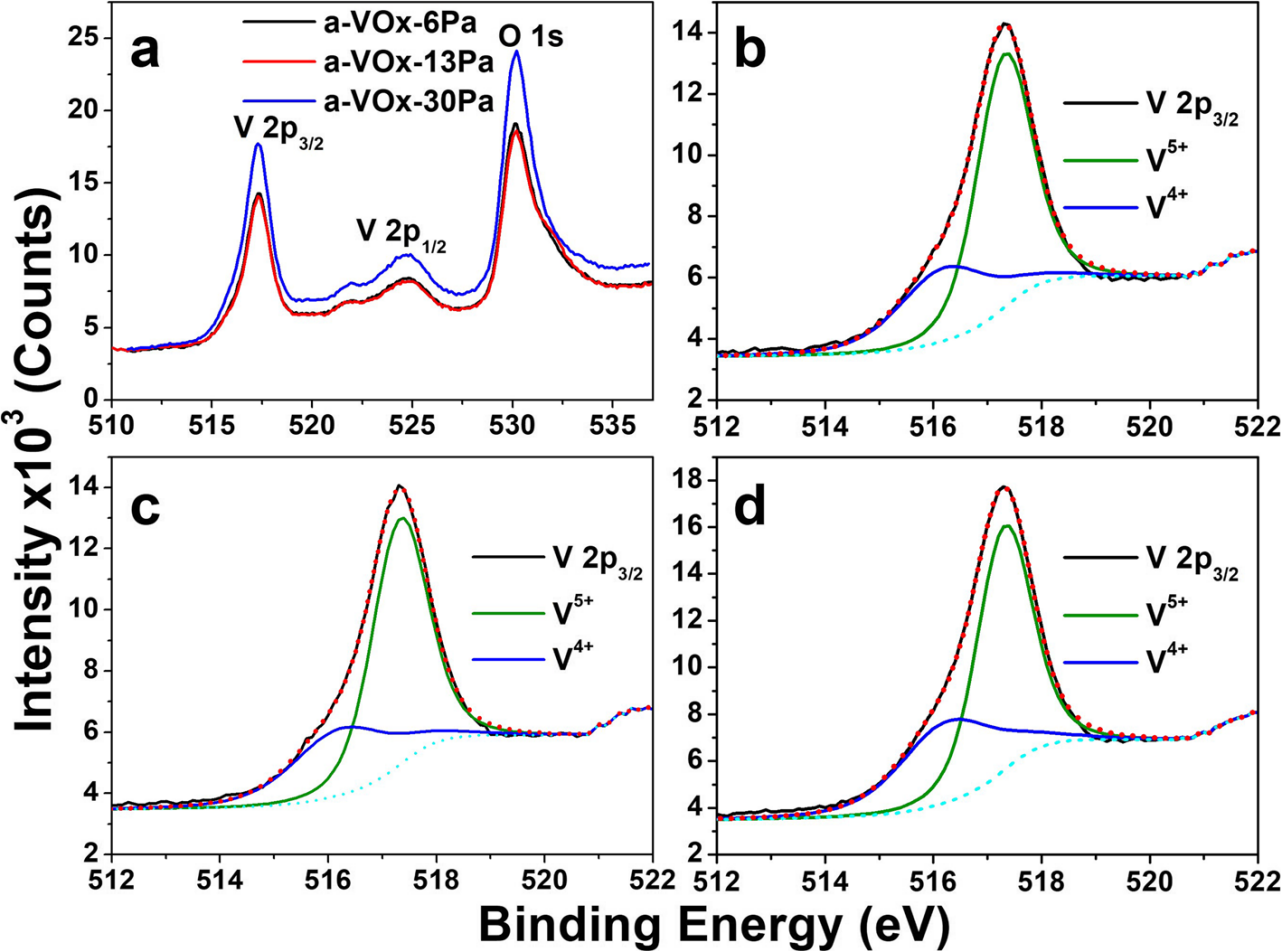
High-resolution XPS spectra of a-VOx films (**a)** O 1s and V 2p core level; V 2p_3/2_ of (**b**) a-VOx-6 Pa, (**c**) a-VOx-13 Pa and (**d**) a-VOx-30 Pa

High-resolution XPS spectra of a-VOx-6, a-VOx-13 and a-VOx-30 Pa films that comprised of characteristic V 2p doublet and O 1s core level peaks are shown in Fig. 3. The features and positions of each peak superimpose on each other in the three cases with slight variation in intensity counts. The peaks of V2p_3/2_, V2p_1/3_ and O 1s are found to be centred at 517.30, 524.8 and 530.2 eV, respectively, and are in good agreement with the reported work [6, 10, 11]. Among the three films, a-VOx-30 Pa film possesses slightly higher mass than the other two, and hence, its spectrum showed higher intensity counts (Fig. 3a. Films a-VOx-6 Pa and a-VOx-13 Pa have shown the same intensity counts that is in strong agreement with nearly equal V/O atomic ratio observed in EDAX findings. A close examination of V2p_3/2_ region revealed a substantial difference in these three films as listed in Table 2. Each V 2p_3/2_ spectrum is further deconvoluted into two peaks corresponding to V^5+^ (green) and V^4+^ (blue) oxidation states (Fig. 3b–d). In all three cases, At% of V^5+^ (~ 68–64%) is found greater than V^4+^ (~ 32–36%). As listed in Table 2, At% of V^5+^ continuously decreased with increasing pO_2_. At% of V^5+^ is noticed to decrease about 0.35% and 4.7% when pO_2_ increased from 6 to 13 Pa and 6 to 30 Pa, respectively. The changing trend in At% of V^4+^ is found exact opposite and equal to V^5+^ oxidation state. Therefore, higher pO_2_ environment say above 13 Pa seems to create more V^4+^ state, i.e. more oxygen deficiency. These observed changes in At% are well consistent with O/V atomic ratio variations noticed in EDAX analysis. Thus, overall XPS analysis is well complementing with the EDAX inferences.

**Table 2:** XPS analysis of V 2p_3/2_ in a-VOx thin films

Name	Oxidation state	Position (eV)	FWHM (eV)	At%
a-VOx-6 Pa	V^5+^ 2p_3/2_	517.31	1.21	68.35
	V^4+^ 2p_3/2_	516.20	1.98	31.65
a-VOx-13 Pa	V^5+^ 2p_3/2_	517.32	1.22	68.00
	V^4+^ 2p_3/2_	516.23	2.10	32.00
a-VOx-30 Pa	V^5+^ 2p_3/2_	517.31	1.21	63.65
	V^4+^ 2p_3/2_	516.27	2.08	36.35

### Electrochemical Characterization

#### Li-Ion Battery Results

**Fig. 4:**
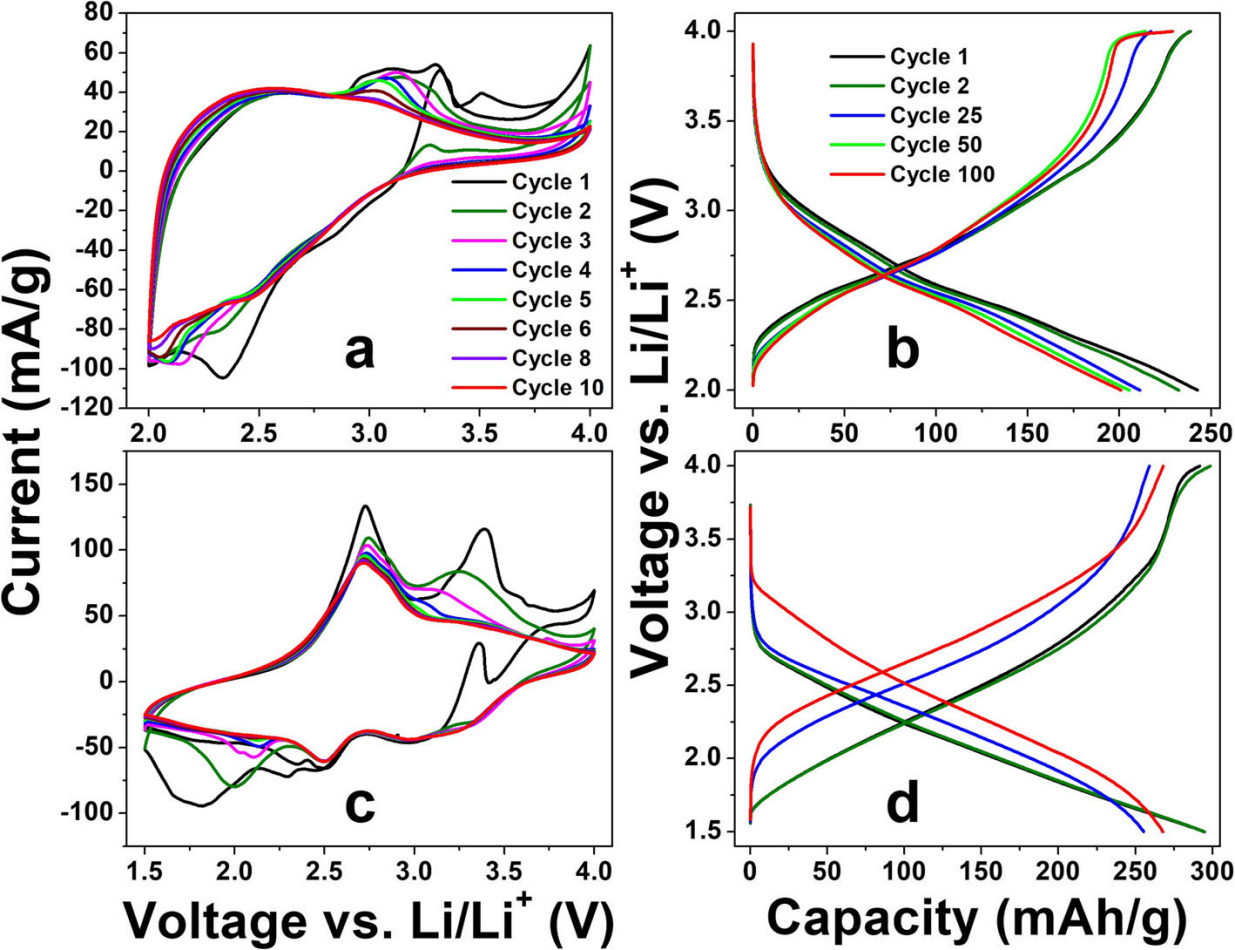
Li-ion battery CV curves (**a**, **c**) and GC profiles at 0.1 C (**b**, **d**) of a-VOx-6 Pa

**Fig. 5:**
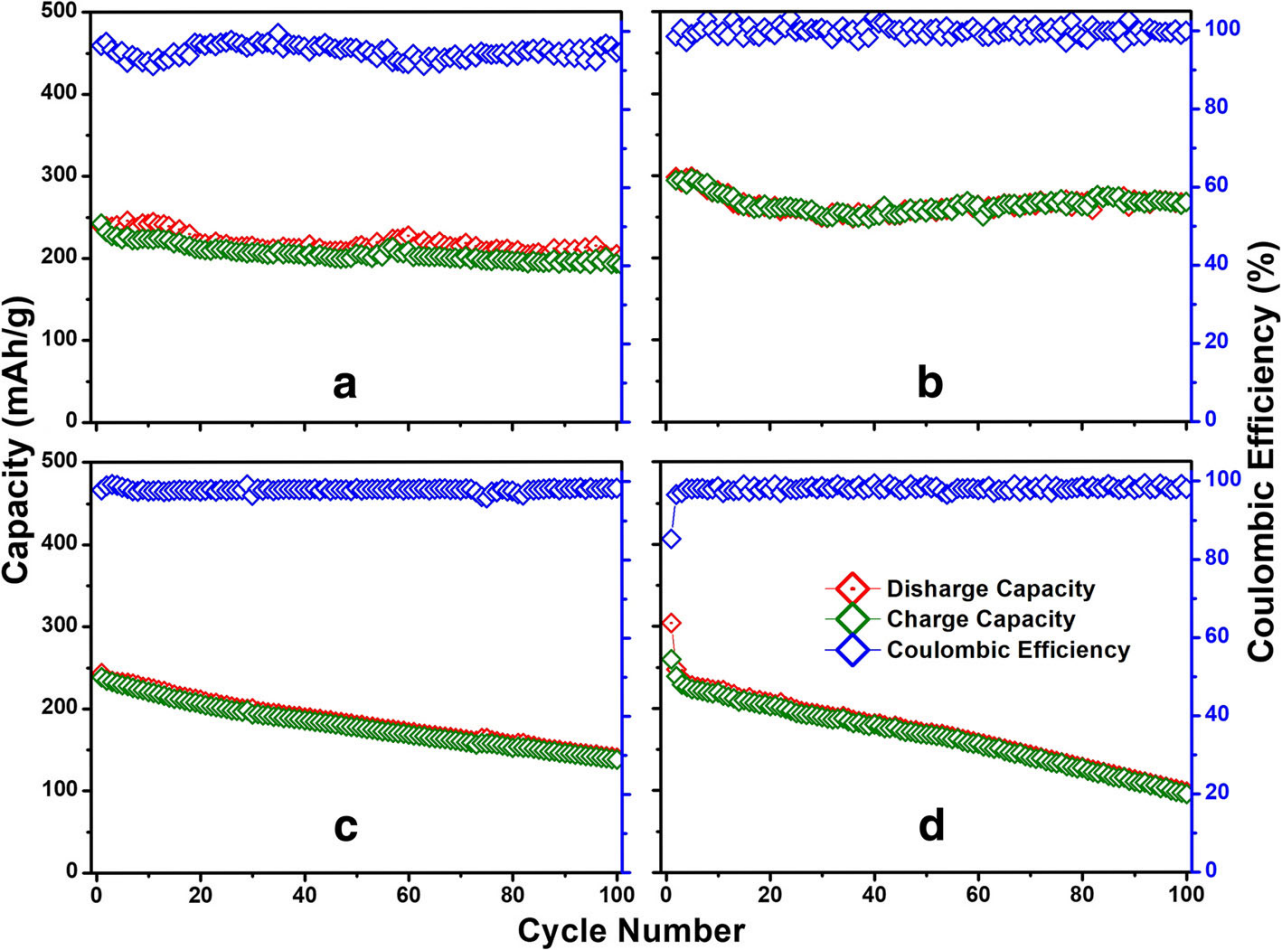
Li-ion battery cyclic performance of a-VOx-6 Pa (**a**, **b**) in comparison with bulk c-V_2_O_5_ (**c**, **d**) in the voltage windows 2.0–4.0 V (**a**, **c**) and 1.5–4.0 V (**b**, **d**)

**Fig. 6:**
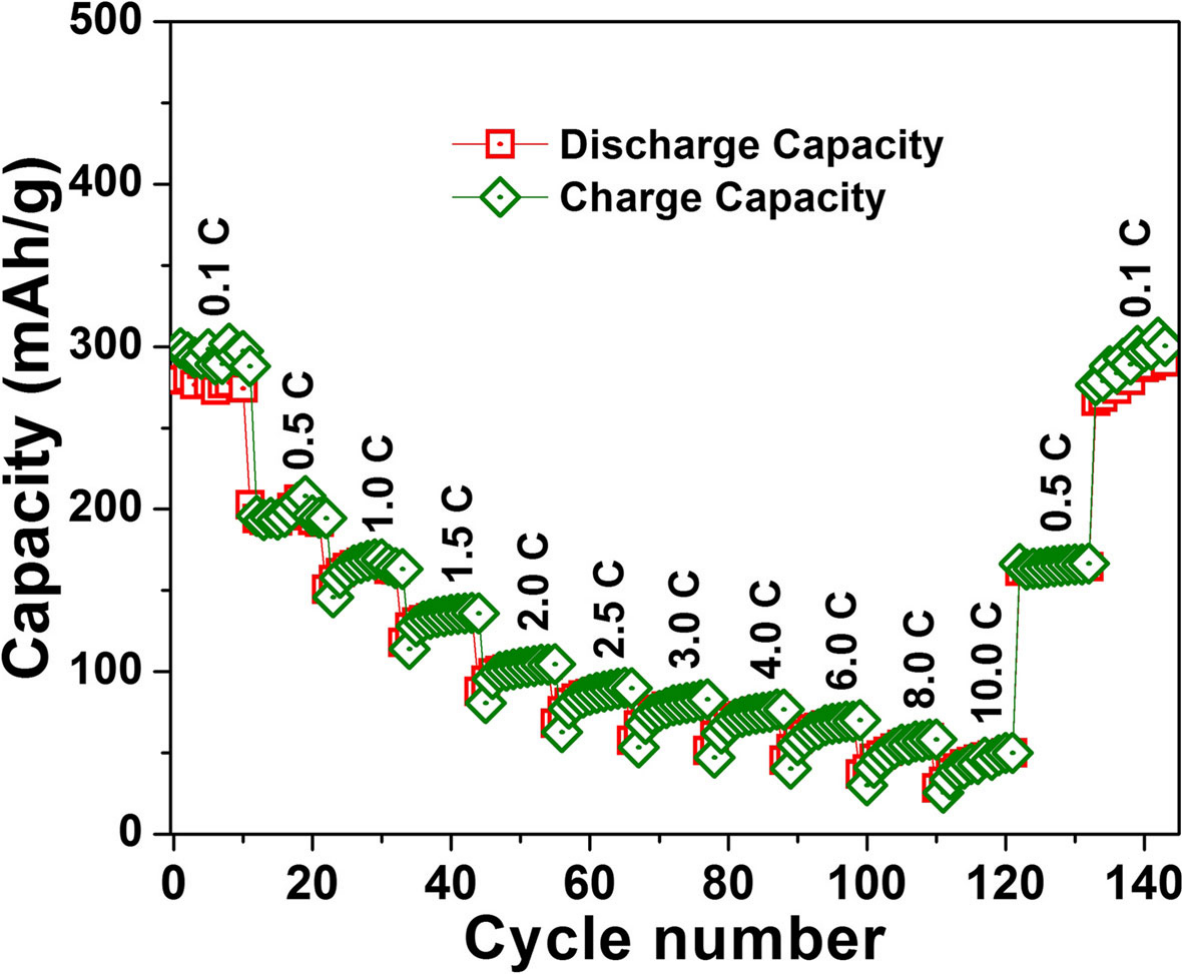
Li-ion battery rate performance of a-VOx-6 Pa

The LIB test results of as-deposited a-VOx-6 Pa, a-VOx-13 Pa and a-VOx-30 Pa films are shown in Fig. 4 and Additional file 1: Figure S13. Amongst the three, the a-VOx-6 Pa films demonstrated excellent performance with high reversible capacities and stable cycling in both voltage windows 2.0–4.0 and 1.5–4.0 V. The CV characteristics of a-VOx-6 Pa resembled pseudocapacitive behaviour in both voltage windows (Fig. 4a, c). Fast faradaic reactions due to lithium’s surface adsorption and desorption process are mainly responsible for the pseudocapacitive behaviour [10]. Here, irreversible current peaks observed up to the fifth cycle are mainly originated from the stainless steel substrate (Additional file 1: Figure S14) with least contribution from electrolyte decomposition that leads to solid electrolyte interphase (SEI) formation. Li-ion (de)intercalation signatures are totally absent when compared to bulk crystalline V_2_O_5_ as shown in Additional file 1: Figure S15. The absence of the intercalation features confirms the complete amorphous nature in tune with the XRD inferences. The observed GC charge-discharge profiles obtained after mitigating substrate effect (Fig. 4b, d) are consistent with CV results. A small charging capacity plateau around 4 V is evolved and disappeared when cycled in-between 2.0 and 4.0 V (Fig. 4b) as a consequence of continuous modifications of vanadium local environment to acquire more symmetry for lithiation [5]. This plateau became much smaller for 1.5–4.0 V cycling, but midpoint of capacity curves shifted towards higher voltage which is an indication for increased ionicity of the matrix. Such increase in ionicity might originate from vanadium reduction to V(III) in addition to local vanadium-oxygen coordination changes, i.e. further amorphisation [5, 27–29]. Overall charge-discharge features of a-VOx-6 Pa are similar to other a-VOx materials synthesised by different routes in other works as referred in the present work. During the first cycle at 0.1 C rate, a-VOx-6 Pa showed high reversible capacities of 239 and 298 mAh g^−1^ in voltage windows 2.0–4.0 and 1.5–4.0 V, respectively. At the end of the 100th cycle, nearly 90% of capacity retention is noticed in both the voltage windows (Fig. 5a, b) as mentioned in Table 3. A 100% Coulombic efficiency is observed throughout the 1.5–4.0 V window cycling whereas it slightly fluctuated around 100% in case of 2.0–4.0 V window as a result of continuous changes to the local vanadium-oxygen environment [5]. Further, it has shown good rate capability as shown in Fig. 6 with capacities ranging from 300 to 50 mAh g^−1^ at 0.1 and 10.0 C current rates, respectively. At 1.0 and 10.0 C, it delivered capacities more than 150 and 40 mAh g^−1^, respectively. The observed cycling is far better than the commercial crystalline V_2_O_5_ powder that was tested in a conventional way (Fig. 5c, d); it retained only 38–57% of initial capacity in both the voltage windows. During galvanostatic cycling in the 1.5–4.0 V potential window, a-VOx-6 Pa showed ~ 15% more capacity than crystalline V_2_O_5_ during the first cycle and that difference goes up to 63% at the cycling termination. Furthermore, its performance is found to be superior to some of the reported PLD a-VOx films deposited on the electrochemically active SnO_2_ substrate as listed in Table 4. SnO_2_ is a well-known anode material for LIBs and could deliver a capacity around 150 mAh g^−1^ in-between 1.5 and 3.0 V at a current rate of 100 mA g^−1^ [30, 31]. The performance of a-VOx-6 Pa is comparable even with the advanced ALD films (3.5 nm) at 1 C as shown in rate capability test (Fig. 6). But its performance is found slightly inferior to other CVD and ALD films. In PLD, very harsh ambient conditions might lead to poor quality V-O coordination when compared with CVD or ALD methods in which slow and steady low-temperature deposition conditions may generate high-quality V-O coordinations. Nevertheless, the overall performance of a-VOx-6 Pa is well superior to c-V_2_O_5_ bulk powder and reported PLD a-VOx films. The performance of a-VOx films can be best suitable for space constraint and limited capacity requirement applications and comparable to latest Li-S batteries [32–37] vowing to their unique advantages such as no requirement of binder, carbon additive, simple preparation and promising scope to incorporate them into all solid-state batteries. Furthermore, a-VOx films are free from capacity loses due to irreversible compound formation such as solid electrolyte interface and polysulfide ring structures [32–37].

**Table 3:** Li-ion battery cycling performance of a-VOx films and c-V_2_O_5_ at the 0.1 C current rate

Name	Voltage (V)	1st charge capacity (mAh g^−1^)	100th charge capacity (mAh g^−1^)	Capacity retention at 100th charge (%)
a-VOx-6 Pa	2.0–4.0	239	215	90
a-VOx-6 Pa	1.5–4.0	298	268	90
c-V_2_O_5_	2.0–4.0	243	139	57
c-V_2_O_5_	1.5–4.0	260	119	46
a-VOx-13 Pa	1.5–4.0	286	225	79
a-VOx-30 Pa	1.5–4.0	230	55	24

**Table 4:** Li-ion battery performance comparison of a-VOx-6 Pa films with other its class of materials

Reference	Film thickness (nm)	Current rate	Voltage range (V)	Capacity (mAh g^−1^)		Remarks
				Cycle 1	Cycle 100	
Li-ion battery						
a-VOx-6 Pa	650	0.1 C	2.0–4.0	239	215	
a-VOx-6 Pa	650	0.1 C	1.5–4.0	298	268	
1	310	0.1 mA cm^−2^	1.5–4.1	390*	346	SnO_2_ substrate
	150		2.0–4.1	240*	193	
2^#^	–	0.2 C	1.8–4.1	330*	240*	
4	450	0.1 C	1.5–4.0	290*	275	ALCVD
5	30	1.0 C	1.5–4.0	356	310*	ALD
7	3.5	1.0 C	2.75–3.0	65	–	ALD
Na-ion battery						
a-VOx-6 Pa	650	0.1 C	1.5–4.0	128	118	
a-VOx-13 Pa	650	0.1 C	1.5–4.0	162	136	
8	–	0.1 C	1.5–3.8	216	140*	Electrodeposition
9	–	0.17 C	1.0–4.0	220*	–	

^#^Plasma enhanced CVD (PECVD)

*Values deduced from images unless otherwise clearly claimed by authors

^−^Not available

#### Na-Ion Battery Results

**Fig. 7:**
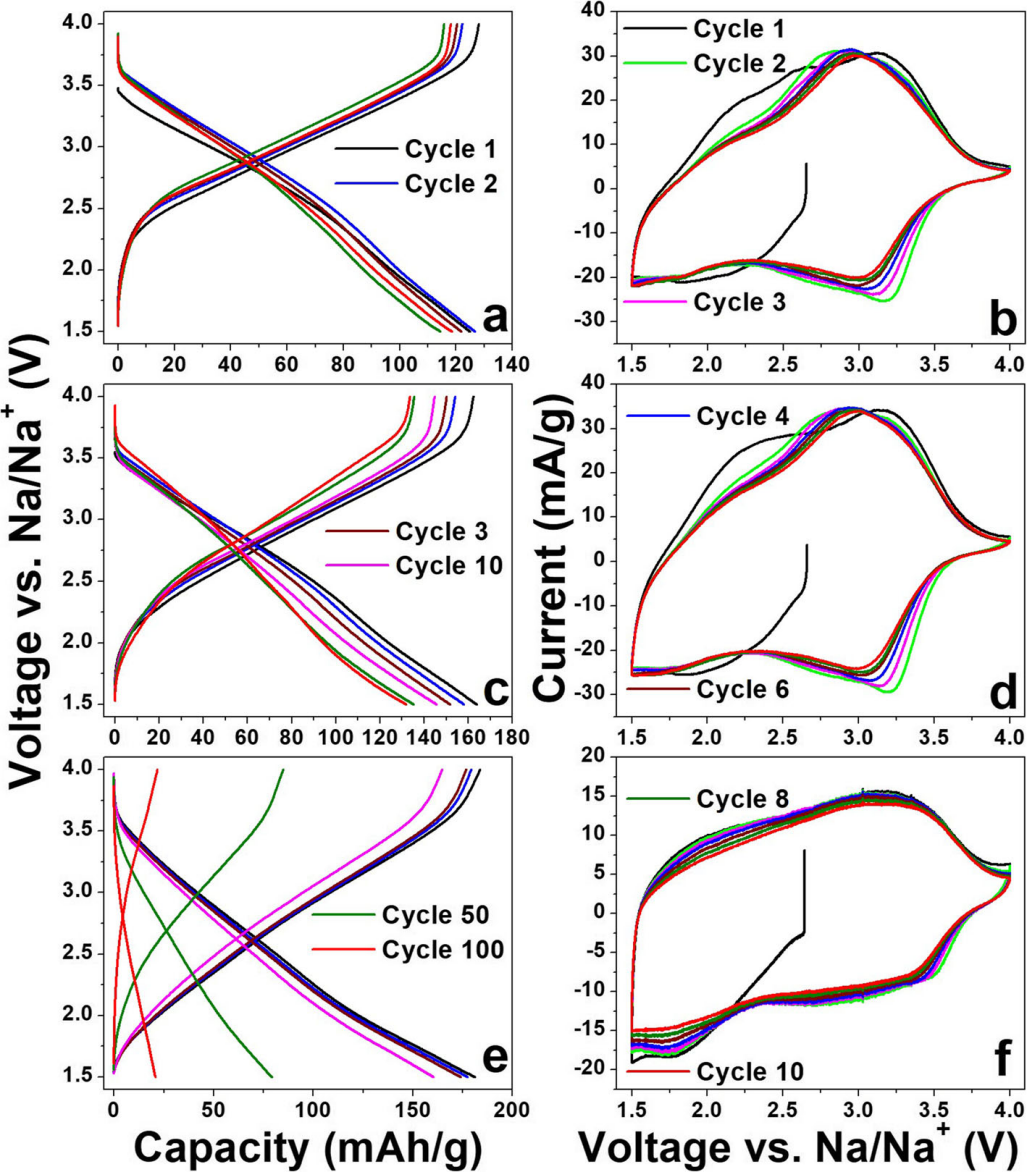
Na-ion battery GC profiles of (**a**) a-VOx-6 Pa, (**c**) a-VOx-13 Pa and (**e**) a-VOx-30 Pa at 0.1 C and respective CV curves shown in (**b**), (**d**) and (**f**)

**Fig. 8:**
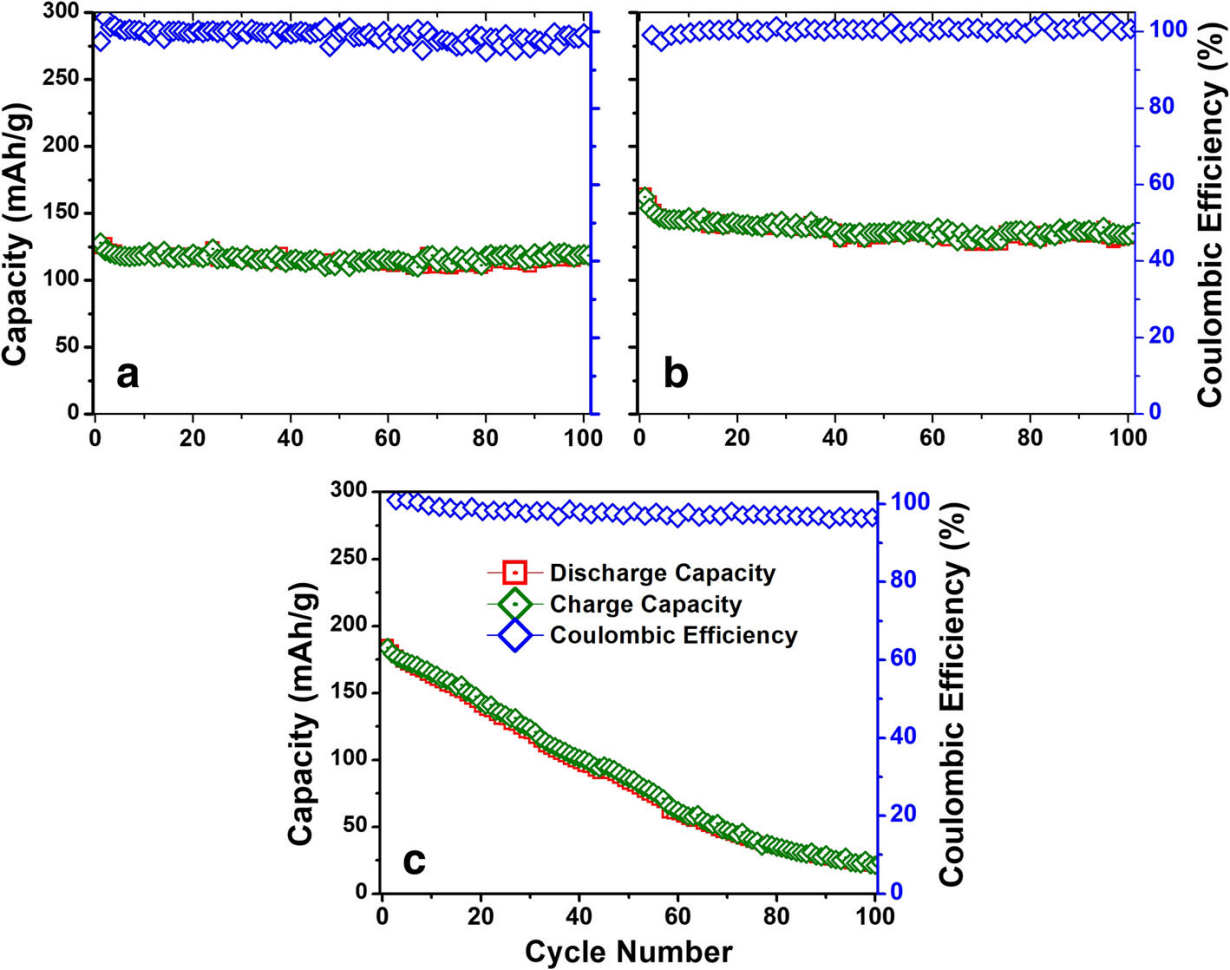
Na-ion battery cycling performance of (**a**) a-VOx-6 Pa, (**b**) a-VOx-13 Pa and (**c**) a-VOx-30 Pa films at 0.1 C

Sodium-ion battery characteristics of a-VOx films are shown in Fig. 7. The GC and CV profile features are found in accordance with each other. Especially CV profiles showed profound pseudocapacitance features with a larger area under the curves than the LIB profiles. Here, no stainless steel peaks appeared, unlike the LIBs case. Out of the three films, a-VOx-30 Pa showed a continuous decrease in CV current whilst the other two stabilized from the fifth cycle onward. Even after the 100th cycle, the similar trend continued as shown in Additional file 1: Figure SI6. The continuous decrease in CV current together with the change of charge-discharge midpoint to a higher voltage is a combined evidence for continuous amorphization of a-VOx-30 Pa film [5, 27–29]. The GC profiles of the three cases are found to be similar, but their charge-discharge crossover points are slightly higher than the respective LIB counterparts. The continuous sloped charge-discharge curve features are the consequence of pseudocapacitance behaviour that originates from fast faradaic surface reactions occurring at cavities and disorders in the cathode matrix [10]. The initial capacities as enumerated in Table 5 are noted to increase with increasing pO_2_. We speculate that this effect might plausibly arise from increased porosity like increased surface roughness observed in the AFM and FESEM analyses that intake more sodium. The absence of such increasing capacity trend in LIBs cycling suggests the type of cavities and porous environment formed best suits for Na-ion insertion with current electrolyte selection. It seems that more porosity leads to a high extent surface adsorption-desorption of sodium, i.e. occurring of very fast faradaic reactions as seen in Fig. 7f. Here, CV curves formed an almost rectangular profile which is very close to a pure electric double-layer capacitor nature [38]. This increased porosity effect is advantageous for only the initial few cycles as capacity continuously faded over the course of cycling as shown in Fig. 7e and Fig. 8c. Continuous SEI formation due to high oxygen-deficient (>V4+) environment might be one of the many reasons to the observed capacity fading that imposes more and more insulating nature to electrode matrix, i.e. losing electrical contacts with the current collector. On the other hand, films deposited below pO_2_ ~ 13 Pa exhibited good cycling stability with Coulombic efficiencies fluctuated around 100% as shown in Fig. 8a, b vowing to good V-O coordination as observed in EDAX and XPS analysis. Among these two films, a-VOx-6 Pa showed excellent cycling stability with 90% capacity retention at the end of the 100th cycle even though its capacity is lesser than a-VOx-13 Pa. The deposition at pO_2_ ~ 13 Pa seems to be good for practical application as it enabled the film to deliver a reversible capacity of 162 mAh g^−1^ that can be retained up to 84% at the end of testing. To compare with present investigation findings, there are very few reports available on pristine a-VOx for sodium-ion storage as listed in Table 4. For example, electrodeposited a-VOx exhibited high capacity of 216 mAh g^−1^ at 1st and that increased to 241 mAh g^−1^ at 2nd cycle from where it faded continuously to 140 mAh g^−1^ at 100th cycle. This inferior cycling behaviour might have resulted from the presence of 12 wt% residual water moieties even after annealing in vacuum. Compared to this case, a-VOx-13 Pa showed far better cycling stability being free from water molecules. In another similar report, a-VOx electrodeposited on graphite paper exhibited a capacity of 220 mAh g^−1^ during first discharge to 1.0 V. When compared to 1.5 V cutoff, it could deliver about 150 mAh g^−1^ capacity that is comparable to a-VOx-13 Pa. In addition, information about the effect of water species that was left either in a-VOx or in graphite substrate was not mentioned. Hence, electrochemistry of PLD a-VOx films is found to be unique and superior to other their kind of materials.

**Table 5:** Na-ion battery cycling performance of a-VOx films and at 0.1 C current rate

Name	Voltage (V)	1st charge capacity (mAh g^−1^)	100th charge capacity (mAh g^−1^)	Capacity retention at 100th charge (%)
a-VOx-6 Pa	1.5–4.0	128	118	92
a-VOx-13 Pa	1.5–4.0	162	136	84
a-VOx-30 Pa	1.5–4.0	184	22	12

#### Electrochemical Impedance Spectroscopy Analysis

**Fig. 9:**
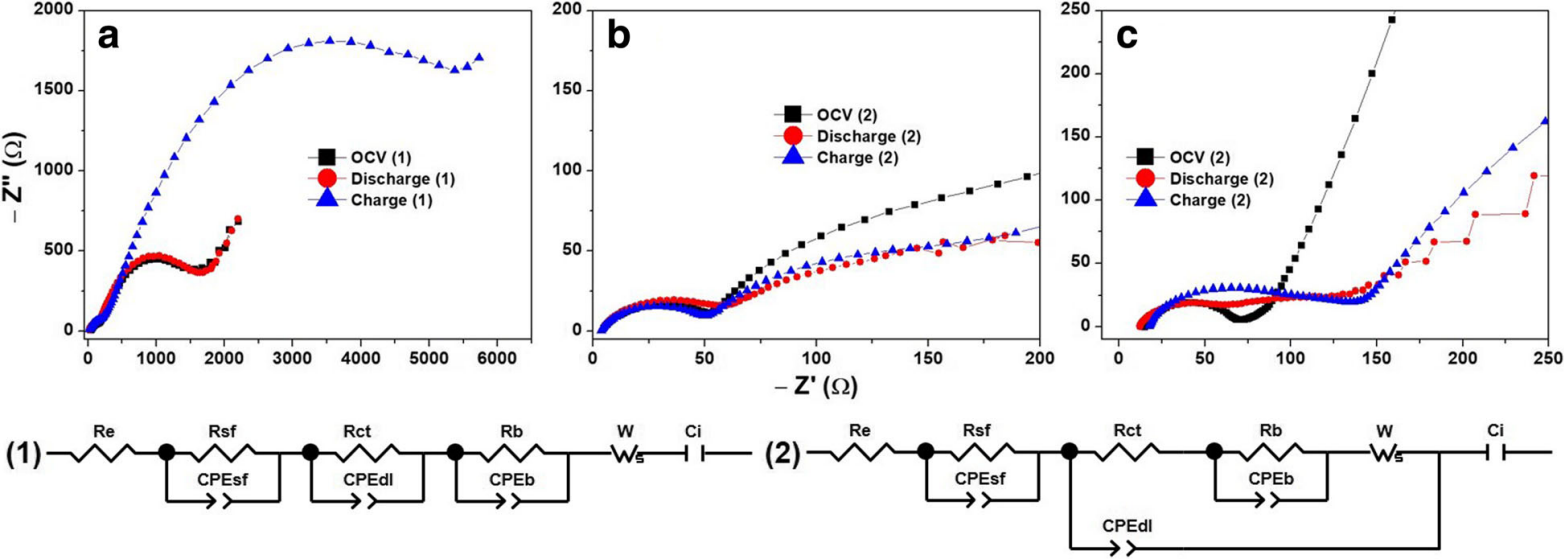
Li-ion battery impedance analysis of a-VOx films deposited at pO2 (**a**) 6 Pa, (**b**) 13 Pa and (**c**) 30 Pa

**Fig. 10:**
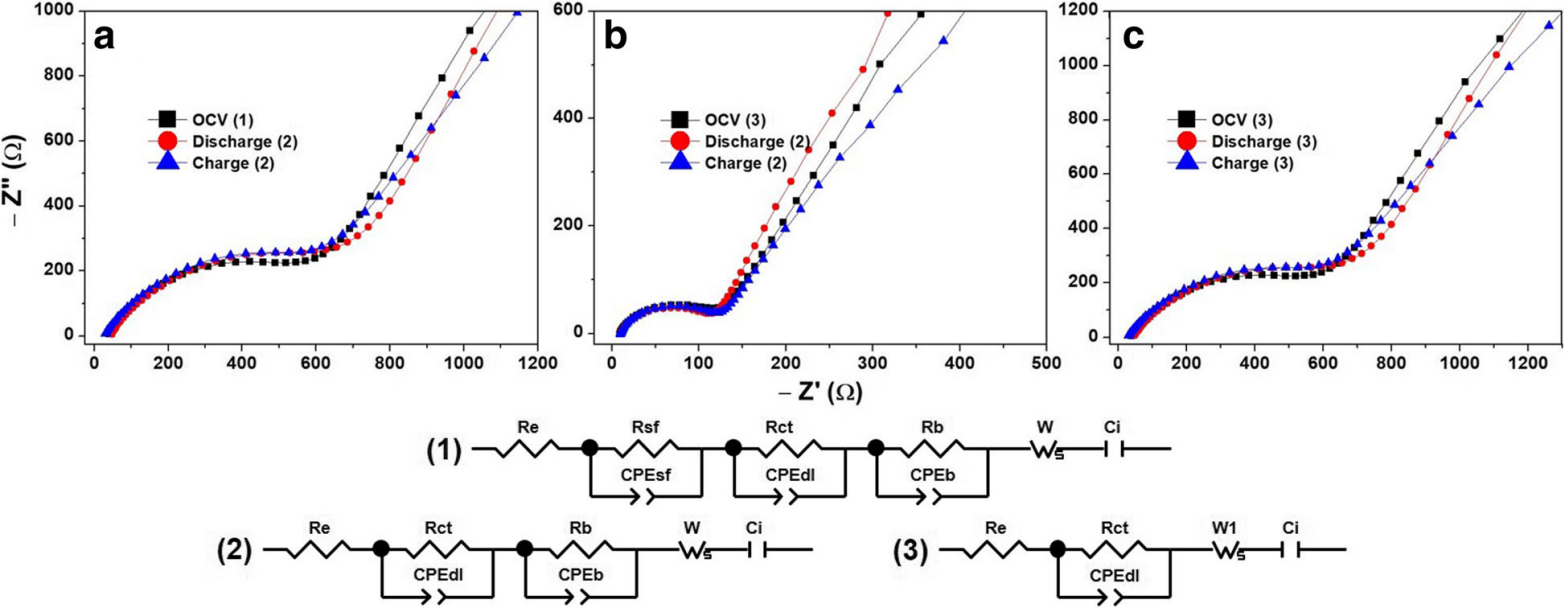
Na-ion battery impedance analysis of a-VOx films deposited at pO2 (**a**) 6 Pa, (**b**) 13 Pa and (**c**) 30 Pa

Lithiation and sodiation kinetics and impedance characteristics of a-Vox films are obtained at open circuit voltage (OCV), discharged to 1.5 V and charged to 4.0 V conditions. The EIS results plotted as Nyquist plots are shown in Fig. 9 for LIBs and Fig. 10 for SIBs. The values of various circuit elements shown in the corresponding equivalent circuits are furnished in Table 6 for LIBs and Table 7 for SIBs. The circuits shown contain electrolyte-electrode contact resistance *R*_e_, resistance due to surface film formation *R*_sf_, resistance to charge transfer *R*_ct_, bulk (substrate) resistance *R*_b_, capacitance components due to surface film formation CPE_sf_, double layer of charge formation CPE_dl_, bulk phase CPE_b_ and ionic intercalation *C*_i_ [31, 39–44]. In case of LIBs cycling, *R*_sf_ and *R*_ct_ values varied as 13 Pa < 30 Pa < 6 Pa whilst CPE_dl_ value varied as 30 Pa < 6 Pa < 13 Pa. The very low CPE_dl_ values and 2.5-fold decrease in *C*_i_ (after the first charge) of a-VOx-30 Pa can explain its inferior and rapidly decaying capacity values. In all three cases, *R*_b_ is found greater than *R*_ct_ that indicates lithiation by fast faradaic surface reactions whose rate seems to be slow down with increasing pO_2_ as the difference between *R*_b_ and *R*_ct_ is decreasing [40, 42]. The decreasing *R*_sf_, consistent *R*_ct_ and very large difference between *R*_b_ and *R*_ct_, i.e. very fast lithiation reactions of a-VOx-6 Pa, enabled it to outperform the other two films. On the other hand, during the SIBs cycling, *R*_ct_ values varied randomly, but a-VOx-13 Pa film has lower values than the other two films. Further, it possesses similar and consistent *R*_ct_, *R*_b_, CPE_dl_, CPE_b_ and *C*_i_ during the first discharge and charge. Thus, a-VOx-13 Pa film achieved superior capacity stable cycling performance than the other two films. Therefore, the overall impedance analysis is in good agreement with the cycling features.

**Table 6:** Li-ion battery impedance analysis of a-VOx films

Film	State	*R*_sf_ (Ω)	*R*_ct_ (Ω)	*R*_b_ (Ω)	CPE_sf_ (μF)	CPE_dl_ (μF)	CPE_b_ (μF)	*C*_i_ (mF)
a-VOx-6 Pa	OCV	198.1	415.5	4369	20.6	444.2	285.9	54.2
	1.5 V	158.4	451.6	4483	34.4	265.9	283.3	78.3
	4.0 V	144.4	433.5	4838	45.2	155.0	272.7	12.2
a-VOx-13 Pa	OCV	39.6	16 × 10^−6^	122.3	8.6	723.6	388.3	40.6
	1.5 V	44.6	2.2 × 10^−6^	130	10.1	731.0	259.2	39.5
	4.0 V	50.5	2.0 × 10^−6^	148.5	17.2	840.6	70.0	42.4
a-VOx-30 Pa	OCV	43.8	11.2	16.5	9.2	3 × 10^−6^	315.2	75.0
	1.5 V	76.0	6.6	43.6	30.7	1 × 10^−4^	196.5	149.5
	4.0 V	64.8	15.3	41.0	4.5	1 × 10^−3^	47.1	58.3

**Table 7:** Na-ion battery impedance analysis of a-VOx films

Film	State	*R*_sf_ (Ω)	*R*_ct_ (Ω)	*R*_b_ (Ω)	CPE_sf_ (μF)	CPE_dl_ (μF)	CPE_b_ (μF)	*C*_i_ (mF)
a-VOx-6 Pa	OCV	311	280.4	7769	99.1	175.3	293.4	0.002
	1.5 V	–	522.3	12,446	–	5.2	75.3	0.57
	4.0 V	–	209.1	12,520	–	5.8	100.5	0.54
a-VOx-13 Pa	OCV	–	106.8	–	–	11.8	–	0.21
	1.5 V	–	111.7	4622	–	26.4	542.1	1.41
	4.0 V	–	111.9	4621	–	26.3	542.0	1.42
a-VOx-30 Pa	OCV	–	297.8	–	–	7.3	–	8.2
	1.5 V	–	450.0	–	–	28.8	–	15.8
	4.0 V	–	273.3	–	–	7.6	–	4.6

## Conclusions

We successfully investigated the electrochemical properties of pristine a-VOx films as cathodes in Li- and Na-ion batteries. The growth of a-VOx thin films by PLD as a function of varying pO_2_ is probed systematically with a multitude of characterization techniques. At selected pO_2_ of 0, 6, 13 and 30 Pa, O/V atomic ratios of the films were found to be 0.76, 2.13, 2.25 and 2.0, respectively. Vanadium in the films a-VOx-6, a-VOx-13 and a-VOx-30 Pa is found in 5+ and 4+ oxidation states with a tendency of later state increased as pO_2_ rises. Amorphous VOx films obtained at pO_2_ ~ 6 and 13 Pa found superior to other counterparts for cathode application in Li- and Na-ion batteries with reversible capacities as high as 300 and 164 mAh g^−1^ at 0.1 C current rate, respectively. High Coulombic efficiencies around 100% are noticed throughout the cycling. At the end of the 100th cycle, nearly 90% of capacity retention is noticed in both cases. The observed cycling trend suggests that the (V^5+^) stoichiometric nature of a-VOx is better than the electrochemistry. The superior performance of a-VOx-6 Pa vs. Li and a-VOx-13 Pa vs. Na cycling is aided from low-resistance charge transfer and fast faradaic surface reactions.

## Additional file

Additional file 1: **Figure S11.** SEM images of bare 304 SS. **Figure S12.** XRD patterns of a-VOx films deposited under different pO2 conditions in comparison with 304 SS. **Figure S13.** GC profiles of a-VOx-13Pa (a, b) and a-VOx-30Pa (c, d) at 0.1 C. **Figure S14.** Li-ion battery CV profile of bare 304 SS at 0.1 mV s^−1^. **Figure S15.** Li-ion battery CV (a and c) at 0.1 mV s^−1^ and GC at 0.1 C profiles of crystalline V2O5 (b and d). **Figure S16.** Na-ion battery CV profiles about 10 cycles of a-VOx films at 0.1 mV s^−1^ after 100th GC cycling. (DOCX 1728 kb)

## Abbreviations

AFM: Atomic force microscope
a-VOx: Amorphous vanadium oxide
CV: Cyclic voltammetry
DEC: Diethyl carbonate
EC: Ethylene carbonate
FESEM: Field emission scanning electron microscopy
LIB: Lithium-ion battery
PC: Propylene carbonate
PLD: Pulsed laser deposition
SEI: Solid electrolyte interface
SIB: Sodium-ion battery
XPS: X-ray photoelectron spectroscopy
XRD: X-ray diffraction
