# Supplementary material for: Amorphous Vanadium Oxide Thin Films as Stable Performing Cathodes of Lithium and Sodium-Ion Batteries
Source: Nanoscale Res Lett. 2018 Nov 14;13:363. doi: 10.1186/s11671-018-2766-0 (PMC6235769; doi:10.1186/s11671-018-2766-0)
Supplement: Supplementary file 1 — Figure S11. SEM images of bare 304 SS. Figure S12. XRD patterns of a-VOx films deposited under different pO2 conditions in comparison with 304 SS. Figure S13. GC profiles of a-VOx-13Pa (a, b) and a-VOx-30Pa (c, d) at 0.1 C. Figure S14. Li-ion battery CV profile of bare 304 SS at 0.1 mV s−1. Figure S15. Li-ion battery CV (a and c) at 0.1 mV s−1 and GC at 0.1 C profiles of crystalline V2O5 (b and d). Figure S16. Na-ion battery CV profiles about 10 cycles of a-VOx films at 0.1 mV s−1 after 100th GC cycling. (DOCX 1728 kb) [file 11671_2018_2766_MOESM1_ESM.docx]

**Amorphous Vanadium Oxide Thin Films as Stable Performing Cathodes of Lithium and Sodium Ion Batteries**

Shaikshavali Petnikota ^#, ^[[1]](#footnote-1)^*^, Rodney Chua ^#^, Yang Zhou ^#^, Eldho Edison and Madhavi Srinivasan**^^[[2]](#footnote-2)^†^**

School of Materials Science and Engineering, Nanyang Technological University, Singapore 639798.

**Supporting Information (SI)**

**SI1: SEM imaging of 304 Stainless Steel (SS)**

**
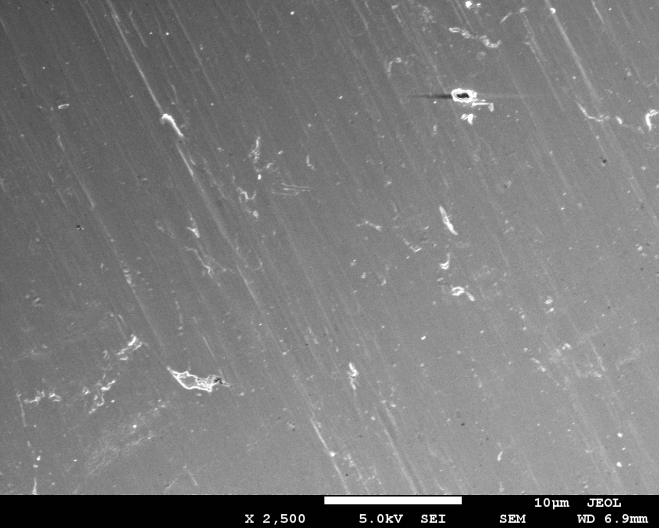

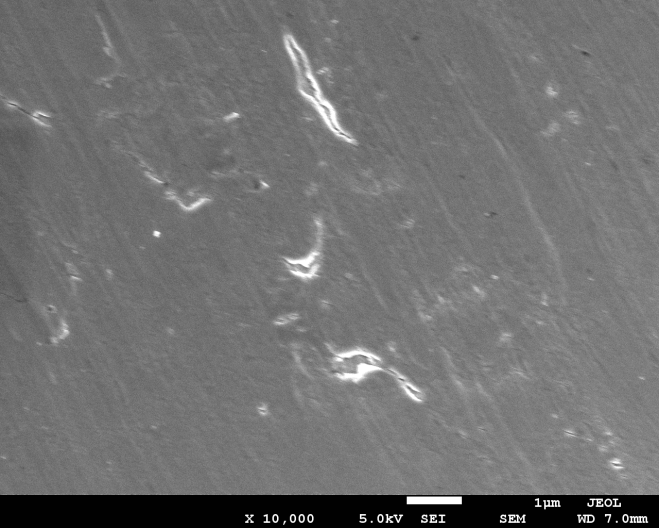
**

**Fig. SI1: SEM images of bare 304 SS.**

**SI2: XRD analysis of a-VOx and 304 SS**

**
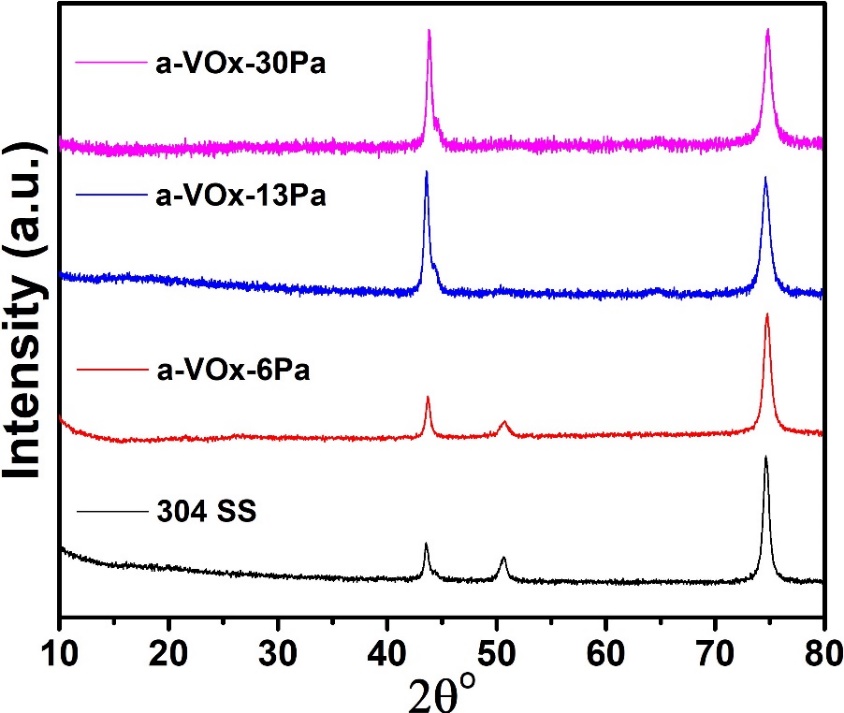
**

**Fig. SI2: XRD patterns of a-VOx films deposited under different pO_2_ conditions in comparison with 304 SS**

**SI3:** **Li-ion battery GC profiles of a-VOx-13Pa and a-VOx-30Pa**

**
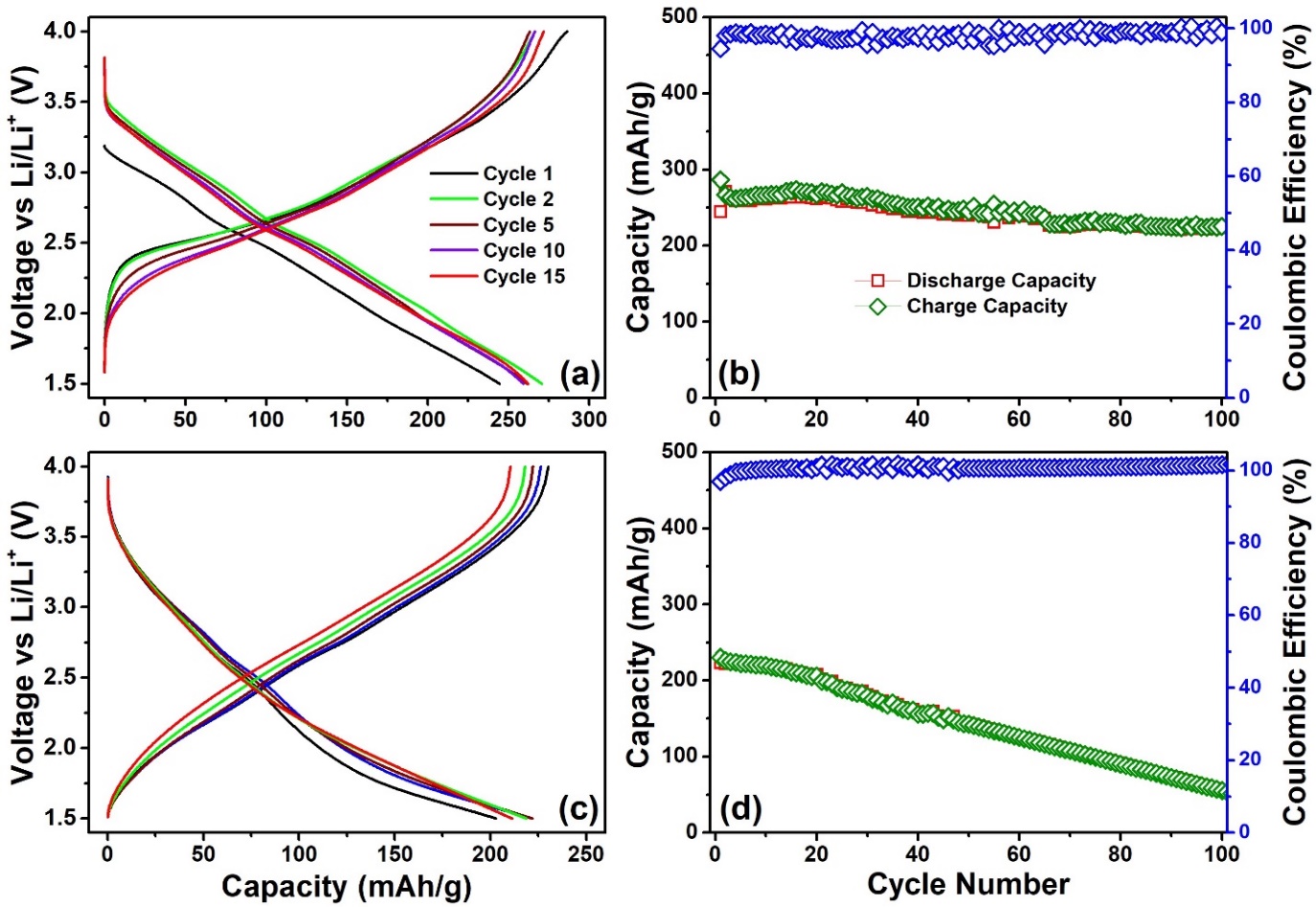
**

**Fig. SI3: GC profiles of a-VOx-13Pa (a, b) and a-VOx-30Pa (c, d) at 0.1 C.**

**SI4: CV of Stainless 304 SS**

**
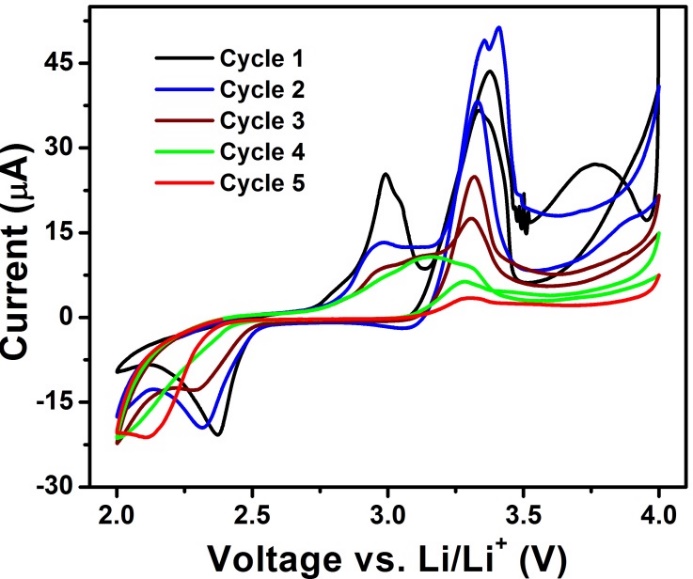
**

**Fig. SI4: Li-ion battery CV profile of bare 304 SS at 0.1 mV s^-1^.**

**SI5:** **Li-ion battery CV and GC profiles crystalline V_2_O_5_**

**
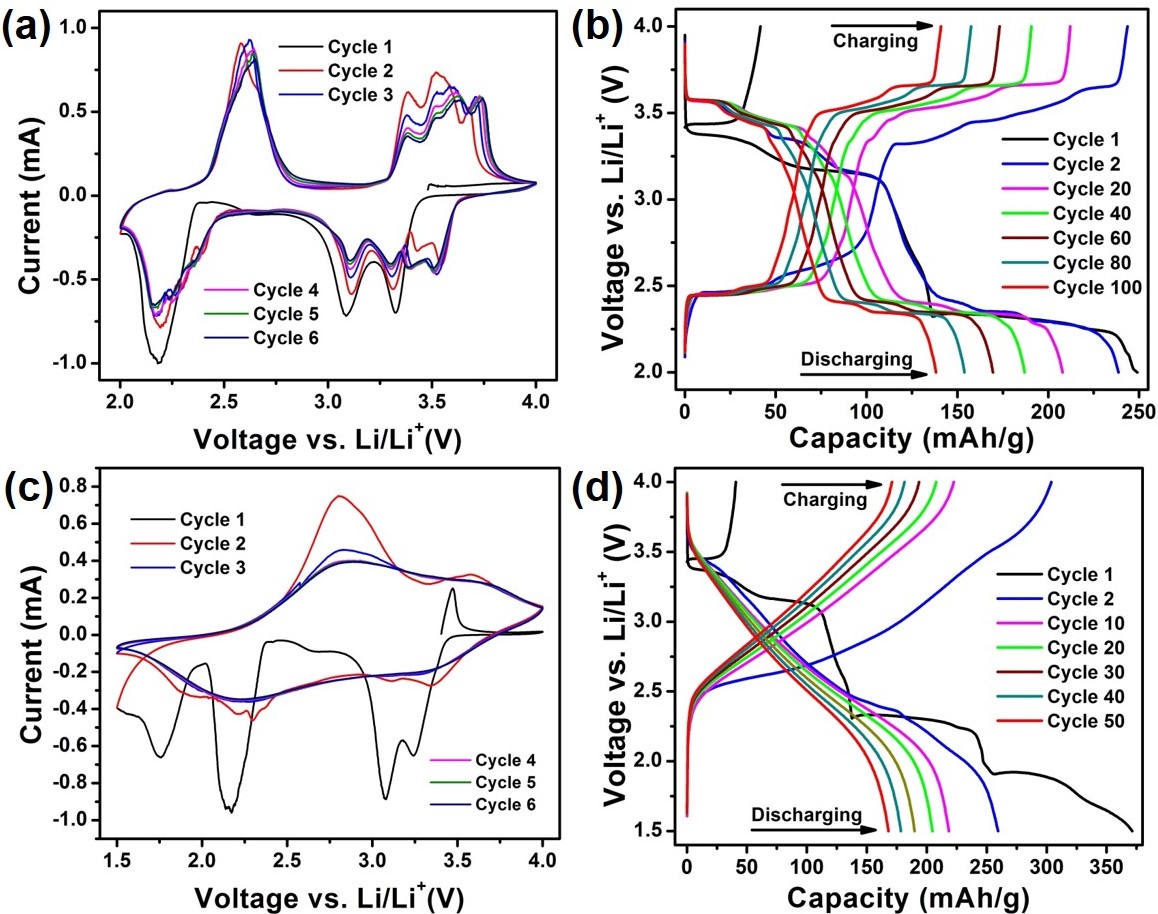
**

**Fig. SI5: Li-ion battery CV (a & c) at 0.1 mV s^-1^ and GC at 0.1 C profiles of crystalline V_2_O_5_ (b & d).**

**SI6: Post-cycling Na-ion battery CVs of a-VOx films**

**
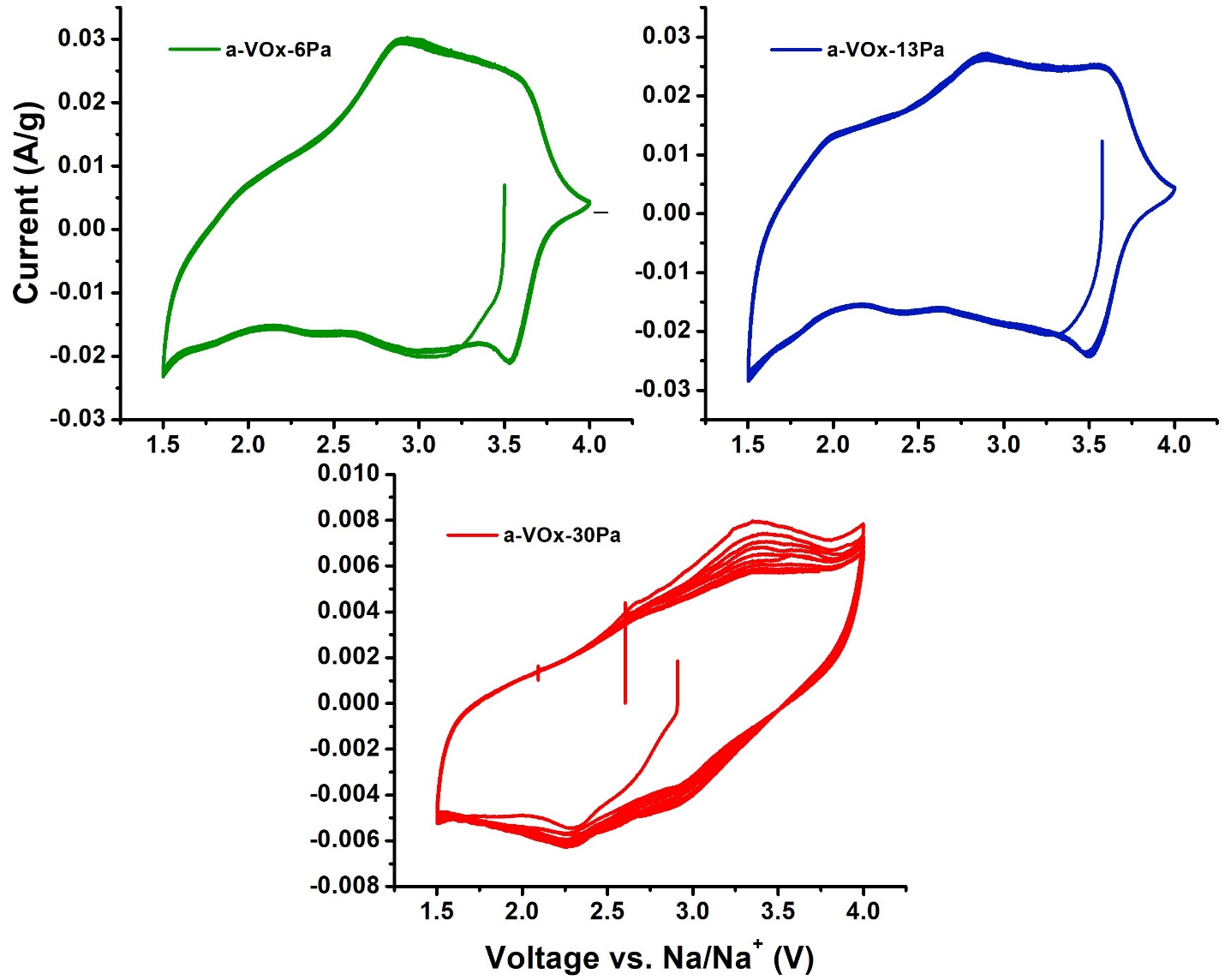
**

**Fig. SI6: Na-ion battery CV profiles about 10 cycles of a-VOx films at 0.1 mV s^-1^ after 100^th^ GC cycling.**

1. ^*^Corresponding author. E-mail address: [pshaikshavali@ntu.edu.sg](mailto:pshaikshavali@ntu.edu.sg); [psvali85@gmail.com](mailto:psvali85@gmail.com) (Shaikshavali Petnikota) [↑](#footnote-ref-1)
2. †Corresponding author. Tel: +65 6790 4606; E-mail address: [madhavi@ntu.edu.sg](mailto:madhavi@ntu.edu.sg) (Madhavi Srinivasan) [↑](#footnote-ref-2)
